# Developing a robust two-step machine learning multiclassification pipeline to predict primary site in head and neck carcinoma from lymph nodes

**DOI:** 10.1016/j.heliyon.2024.e24377

**Published:** 2024-01-12

**Authors:** Jiaying Liu, Anna Corti, Giuseppina Calareso, Gaia Spadarella, Lisa Licitra, Valentina D.A. Corino, Luca Mainardi

**Affiliations:** a Department of Electronics, Information and Bioengineering, Politecnico di Milano, Milan, Italy; b Radiology Department, Fondazione IRCCS Istituto Nazionale dei Tumori di Milano, Milan, Italy; c Postgraduation School in Radiodiagnostics, University of Milan, Italy; d Department of Clinical Medicine and Surgery, Federico II University, Naples, Italy; e Head and Neck Cancer Medical Oncology Department, Fondazione IRCCS Instituto Nazionale dei Tumori di Milano, Milan, Italy; f Department of Oncology and Hemato-Oncology, University of Milan, Italy; g Cardiotech Lab, Centro Cardiologico Monzino IRCCS, Milan, Italy

**Keywords:** Multiclassification, Machine learning, Radiomics, Head and neck carcinoma of unknown primary

## Abstract

This study aimed to develop a robust multiclassification pipeline to determine the primary tumor location in patients with head and neck carcinoma of unknown primary using radiomics and machine learning techniques. The dataset included 400 head and neck cancer patients with primary tumor in oropharynx, OPC (n = 162), nasopharynx, NPC (n = 137), oral cavity, OC (n = 63), larynx and hypopharynx, HL (n = 38). Two radiomic-based multiclassification pipelines (P1 and P2) were developed. P1 consisted in a direct identification of the primary sites, whereas P2 was based on a two-step approach: in the first step, the number of classes was reduced by merging the two minority classes which were reclassified in the second step. Diverse correlation thresholds (0.75, 0.80, 0.85), feature selection methods (sequential forwards/backwards selection, sequential floating forward selection, neighborhood component analysis and minimum redundancy maximum relevance), and classification models (neural network, decision tree, naïve Bayes, bagged trees and support vector machine) were assessed. P2 outperformed P1, with the best results obtained with the support vector machine classifier including radiomic and clinical features (accuracies of 75.3 % (HL), 75.4 % (10.13039/100003224OC), 71.3 % (OPC), 92.9 % (NPC)). These results indicate that the two-step multiclassification pipeline integrating radiomics and clinical information is a promising approach to predict the tumor site of unknown primary.

## 1 Introduction

Head and neck cancer (HNC) is the seventh most common cancer worldwide, accounting for 700,000 annual cases and 350,000 deaths in 2018 [1]. HNC is a highly heterogeneous class of malignancies, and, based on the location of the main tumor, it can be categorized into five main types: laryngeal and hypopharyngeal cancer, nasal cavity and paranasal sinus cancer, nasopharyngeal cancer, oral and oropharyngeal cancer, salivary gland cancer [2]. In 1.5 %–5 % of HNC, metastasis of unknown primary is detected at the lymph node within the head and neck region, but the location of the primary tumor is not known, posing significant challenges in treatment and management [3,4]. In this case, the cancer is known as head and neck carcinoma of unknown primary [3]. The diagnostic protocol to identify the primary occult tumor consists of an initial clinical assessment, including fiberoptic laryngoscopy, followed by computed tomography (CT) and/or magnetic resonance imaging (MRI), cytology and, eventually, positron emission tomography imaging are requested [3]. Nevertheless, if the primary tumor is still occult, a panendoscopy with biopsy is performed at risk hidden tumor sites [3]. Far from being effortless, the diagnostic protocol is complex and extensive, but it is essential to guide the treatment and improve patient survival. In this scenario, developing non-invasive methods to support the forecast of the origin of the unknown primary tumor can be helpful to shorten the diagnostic pathway.

Recently, radiomics, consisting in the extraction and analysis of high-throughput quantitative features from medical images, has emerged as a potential approach for the advancement of cancer precision medicine [5]. In particular, several radiomic studies have been proposed with the aim of contributing to tumor characterization, patient stratification, outcome prediction, biomarkers identification and treatment personalization in HNC patients [6]. However, radiomic studies predicting primary tumor location in HNC patients are lacking. Few studies have identified primary tumor locations of metastases found in lung and brain using radiomics [[7], [8], [9]]. For example, Shang et al. [8] sought to differentiate lung metastases originated from colorectal cancer, breast cancer and renal carcinoma using radiomic features extracted from lung CT images. Ortiz- Ramón et al. [7] examined MRI textures features to distinguish brain metastases originated from lung cancer, melanoma and breast cancer. Both studies applied multiclass machine learning approach with the maximum of three classes to be differentiated. Another study conducted by Kniep et al. [9] also explored the utility of radiomics of brain MRI in prediction of metastatic tumor type, including metastases originated from breast cancer, small cell lung cancer, non-small cell lung cancer, gastrointestinal cancer and melanoma. In Kniep et al.’s research, although their multiclassification problem comprised five primary tumor locations, they built both three-class and five-class models and obtained comparatively poorer results for the five-class model developed. While forementioned studies focused on distinguishing metastases from primary tumors located in distant anatomical regions, our study addresses the challenge of predicting the primary tumor location within the same anatomical region, specifically the head and neck. To the best of the authors' knowledge, only one previous study [10] attempted to predict the primary tumor location of HNC patients with unknown primary using a DNA-based approach, whereas no image-based multiclassification has never been explored.

In this study, we aim to develop a robust and reliable pipeline for multiclass prediction of HNC patients with unknown primary site using radiomics and machine learning techniques. Radiomic features will be extracted from the lymph nodes segmented in MRI of HNC patients with the primary tumor location already diagnosed. The primary locations are oropharynx (OPC), nasopharynx (NPC), oral cavity (OC), and hypopharynx and larynx (HL).

## 2 Methods

### 2.1 Patient dataset

#### 2.1.1 Patient population

Patients included in this study were part of the BD2Decide project (ClinicalTrial.gov Identifier: NCT02832102), a multi-centric clinical study in locally advanced HNC [11]. The conduction of these studies was approved by Institutional Ethical Committee (study protocol identifiers INT65-16 and INT66-16 for the BD2Decide)**,** and data acquisition followed the General Data Protection Regulation of the EU. All methods were carried out in accordance with relevant guidelines and regulations. For data use and retrieval of tumor samples, when available, patients still alive provided informed consent; a waiver for informed consent was obtained according to national regulations. For deceased patients in the retrospective cohort, a waiver was provided by the Institutional Review Board of Istituto Nazionale dei Tumori, Milan, Italy. Study inclusion criteria were: 1) at least 2 years of follow-up for alive patients; 2) availability of at least one spin-echo MR image sequence (either T1-or T2-weighted); 3) images acquired with 1.5 T scanner; 4) absence of magnetic artifacts or movement-related artifacts in the MRI images; 5) visibility of the primary tumor in the MRI.

The dataset analyzed in this study comprised 418 HNC patients collected at National Cancer Institute (Milan, Italy). However, patients belonging to the G3Spinocell group (18 patients) were removed from the following analysis as this tumor can contain more than one site. Thus, 400 patients were included in this study, with their clinical characteristics are shown in Table 1.

**Table 1:** Clinical characteristics of patients included in the study.

Patient characteristics	

**Median follow-up**	40 [25–62] months
**Gender**	
M	197
F	203
**Median age (range)**	56 [18–83]
**Primary tumor site**	
Oral cavity	63
Oropharynx	162
Nasopharynx	137
Hypopharynx and Larynx	38
**AJCC 8th edition**	
I	121
II	35
III	93
IV	151
**Smoking**	
Current/former	228
Never	172
**HPV status**	
Positive	248
Negative	152

#### 2.1.2 Image acquisition, segmentation and preprocessing

T1-weighted (T1w) and T2-weighted (T2w) images were acquired using turbo spin-echo pulse sequences. The region of interest (ROI), corresponding to the largest lymph node, was segmented on each slice by HNC expert radiologists to produce a 3D ROI. Only one ROI was drawn per patient and was segmented using the T2w sequence as a reference, when available. After T2w segmentation, lesion margins were checked and corrected based on other imaging sequences (pre-contrast T1w or, when available, post-contrast T1w or diffusion-weighted images).

To account for the different MRI volume acquisition parameters and the presence of intensity modifications due to speckle noise and local variation of the magnetic field, different steps of image preprocessing were applied [12,13]. First, a 3D Gaussian filter with a 3 × 3 × 3 voxel kernel and σ = 0.5 was used to denoise the images. Then, the N4ITK algorithm [14] was used to estimate and correct intensity non-uniformities due to local variations of the magnetic field. Intensity standardization in each volume was performed using Z-score (removing the mean and dividing by the standard deviation of the grey values) to ensure that each MRI image had similar ranges of signals. Voxel size resampling to an isotropic resolution of 2 mm (as in Leijenaar et al. [15]) was performed with B-spline interpolation. A fixed-bin histogram discretization (32 bins) was used prior to features extraction.

### 2.2 Feature extraction, preprocessing and selection

#### 2.2.1 Feature extraction

Feature extraction was performed using Pyradiomics 3.0 [16]. A total of 1072 radiomic features, 536 per image type (T1w and T2w) were extracted. The 536 features belonged to different categories: shape and size (14 features), first order statistics (18 features), textural (40 features), wavelet (464 features). Textural features were computed using the grey level co-occurrence matrix (GLCM) and the grey level run length matrix (GLRLM). The full list of radiomic features is available in Pyradiomics documentation [16]. A fixed-bin histogram discretization (32 bins) was used prior to features extraction.

#### 2.2.2 Feature preprocessing

Feature preprocessing consisted of stability analysis, removal of highly correlated features and normalization.

To select a stable feature set, a stability analysis was performed by considering a sensibility analysis of features to small changes in the ROI to mimic the effect of inter-reader variability in the segmentation as described in Refs. [12,17]. Features were considered stable if the intra-class correlation coefficient (ICC) was above 0.75 as defined by Koo et al. [18] based on the 95 % confident interval of the ICC estimate. After the stability analysis, 265 radiomic features per patient were considered.

Redundant features were removed using pairwise correlation. When a pair of features had an absolute Spearman correlation coefficient above a fixed threshold only one of the two was kept. In particular, the one with lower mean correlation with all the other *n-2* features was selected. Obviously, the higher the threshold, the fewer features were removed. Three thresholds were considered (correlation ρ > 0.75, 0.80, 0.85), and 241, 233 and 222 features were removed, respectively, thus 24, 32 and 43 features remained available for the successive steps. The summary of radiomic features obtained after each threshold removal can be found in Table S1 of Supplementary Material. Afterwards, the remaining features were normalized by z-score standardization and prepared to undergo feature selection, which were performed on several data partitions.

#### 2.2.3 Feature selection

Feature selection was performed on the training sets obtained from the *Data partition* (described in Section 2.3.1). Five different feature selection techniques were assessed, namely, sequential forwards selection (SFS), sequential backwards selection (SBS), sequential floating forward selection (SFFS), neighborhood component analysis (NCA) and minimum redundancy maximum relevance (MRMR). These methods were chosen based on Zhang et al.’s review [19] and detailed description of the algorithms is provided in the Supplementary Materials.

For each feature selection technique, 30 different feature sets were obtained (one for each training set). Subsequently, the frequency of selection of each feature over the 30 repetitions was evaluated and the most frequently selected ones (according to the empirically chosen thresholds defined below) were included in the final feature set for the model training. Specifically, five different frequency thresholds were considered as inclusion criteria, namely F1, F15, F20, F25, and F30, if the specific feature was selected in at least 1, 15, 20, 25 or in all the 30 features sets, respectively. Finally, for each correlation threshold combined with each of the five feature selection techniques, five sets of features were obtained, for a total of 75 feature sets.

### 2.3 Pipelines

Two multiclassification pipelines were developed and tested, based on a supervised learning approach. The first machine learning pipeline was designed to identify directly the four primary sites (HL, OC, OPC and NPC), whereas the second pipeline was based on a two-step approach: in the first step, the number of classes was reduced by merging two classes (HL and OC) which were then reclassified in the second step. Both pipelines consisted of four stages: (i) Feature preprocessing, (ii) Data partition, (iii) Feature selection, (iv) Classification model development. Steps (i) and (ii) were explained in section 2.2.2, 2.2.3, respectively, since they share the same methodology in both pipelines, while the two other steps, (ii) and (iv) are pipeline specific and they are detailly explained in section 2, 2.3.1.3.2, respectively. To accomplish these steps, Matlab version R2022a was exploited.

#### 2.3.1 Pipeline 1

**Fig. 1:**
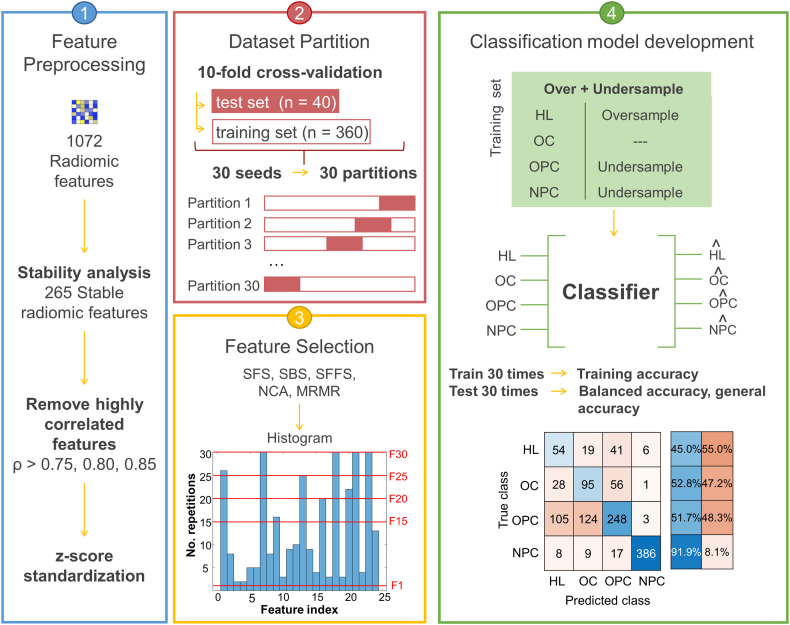
P1 approach. Step 1 shows *feature preprocessing*, where highly correlated features were removed and standardized. Step 2 explains the *data partition* into training and test sets, 30 times differently. Step 3 represents *feature selection*, where five methods were assessed and for each method, a frequency histogram was built. Step 4 constitute the *classification model development*, which includes over- and undersampling the training set, model training to predict tumor locations using the radiomic features selected and subsequently model validation.

The objective of pipeline 1 (P1) was to identify the four primary sites, i.e. HL (n = 38), OC (n = 63), OPC (n = 162) and NPC (n = 137). The stages of P1 are represented in Fig. 1 and details about each step are provided below.

##### 2.3.1.1 Data partition

The dataset was partitioned into training and test sets by considering internal 10-fold cross-validation. To increase robustness, the overall process was repeated 30 times by setting 30 different seeds of the random number generator, thus obtaining 30 different training and test sets.

##### 2.3.1.2 Classification model development

As previously observed, the dataset was imbalanced since the number patients from each class was uneven. Thus, to obtain a more balanced dataset to train the classifiers, the training sets obtained from the *data partition* stage underwent oversampling and undersampling techniques, by considering the number of Oral Cavity (OC) patients as reference (i.e., #57). Accordingly, the HL class was oversampled, by adopting the synthetic minority oversampling technique (SMOTE) algorithm [20], while the NPC and OPC classes were undersampled, by randomly selecting patients to be removed. As a result, a balanced training set containing 57 patients per class was obtained.

To identify an optimal classification model, five machine learning models were trained, namely: neural network (NN), decision tree (DT), naïve Bayes (10.13039/100004395NB), bagged trees (BT) and support vector machine (SVM). However, to select the best performing model without being biased from the feature selection method, only feature sets selected by NCA and MRMR were used, as they are independent from the machine learning algorithm. Once the optimal model was selected, the remaining feature sets were assessed by training the model.

Model performances were evaluated in terms of prediction's balanced and general accuracies and the validation values obtained from the model for each of the 30 partitions were assessed by computing the median value, the first and third quantiles.

#### 2.3.2 Pipeline 2 (P2)

**Fig. 2:**
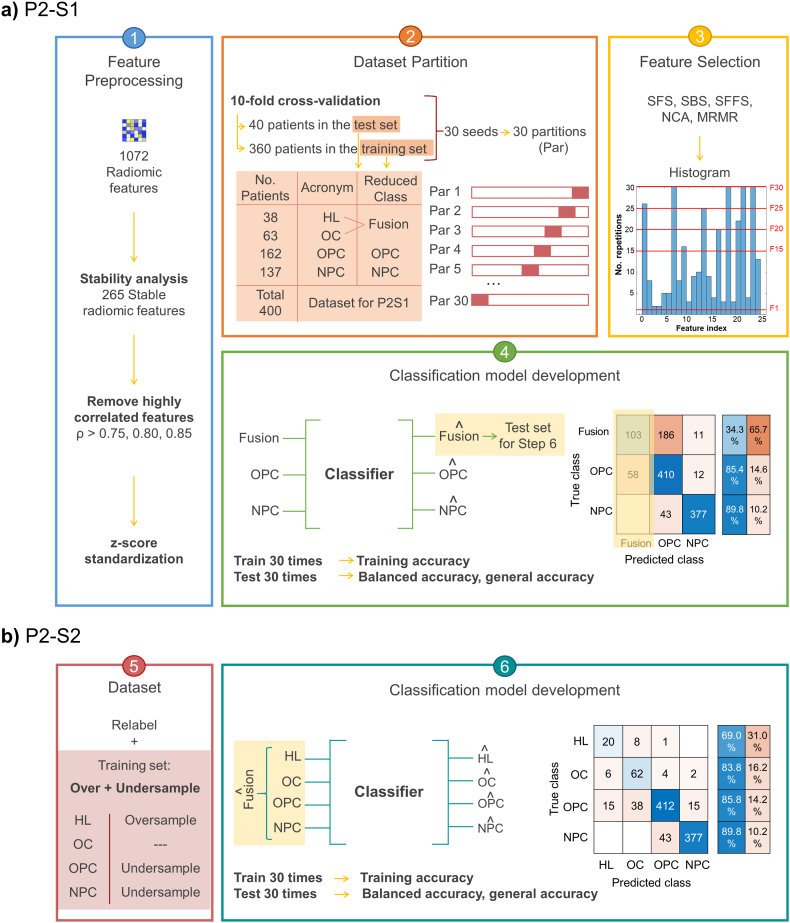
P2 approach: **a)** P2–S1 consists of 1. *Feature preprocessing*, 2. *Data partition*, 3. *Feature Selection* and 4. *Classification model development*. **b)** P2–S2 consists of 5. *Patient relabel and training set over-/undersampling* and, once more, 6. *Classification model development.*

The second pipeline (P2) was designed to simplify the classification task by lowering the number of classes and to solve the imbalanced dataset issue. P2 was a two-step classification approach represented in Fig. 2, the first step will be referred as P2–S1 (Fig. 2a), and the second step as P2–S2 (Fig. 2b). P2–S1 addressed the classification of the majority classes NPC and OPC, while P2–S2 the classification of the minority classes, HL and OC. The same *feature preprocessing* stage adopted for P1, was applied for P2.

In P2–S1 (Fig. 2a), the number of classes was reduced from four to three by combining the two minority classes, HL and OC, into a merged class named "Fusion". The rationale behind merging HL and OC was to obtain a fictional more balanced three-class dataset (i.e., OPC (n = 162), NPC (n = 137) and Fusion (n = 101)) as an intermediate step in our two-step classification approach. Thus, *data partition* was applied on this three-class dataset, and subsequent *feature selection* and *classification model development* stages were performed, with the same methodology explained for P1. Accordingly, with three correlation thresholds, five feature selection methods and five frequency thresholds, a total of 75 models were obtained at the end of P2–S1.

In P2–S2 (Fig. 2b), for each correlation threshold and feature selection method, only one set out of the five feature frequency thresholds (F1, F1, F20, F25, F30) was selected. The selection of the feature set was based on the median value of two metrics listed in order of its importance: balanced accuracy and general accuracy, computed from the models applied on the test sets in P2–S1. When two balanced accuracies obtained from two feature sets selected by the same feature selection methods (after fixing the same correlation threshold) were equal or similar (not exceeding a difference of 1 %), the feature set that corresponded to a greater general accuracy was chosen. For example, fixing 0.80 as correlation threshold and applying NCA as feature selection method, the median value of the balanced accuracy and general accuracy obtained from the model trained using the feature set F20 were 58,0 % and 72,5 %, while using F25 were 57,4 % and 75,0 %. In this case, the balanced accuracies were similar (difference does not exceed 1.0 %) but F25 obtained a higher general accuracy. Therefore, from all the five feature frequency thresholds, F25 was selected to proceed to the second step of this pipeline. In case of same or similar (not exceeding a difference of 1 %) values for both metrics (i.e. balanced accuracy and general accuracy), the feature set with fewer predictors was selected to proceed to P2–S2.

Considering the above, the number of feature sets evaluated in P2–S2 was reduced to 15, i.e., combining 3 correlation thresholds, 5 feature selection methods and 1 frequency threshold. Consequently, a total of 15 feature sets were assessed in the model development of P2–S2. This depletion in the number of feature sets evaluated in the second step is reasonable since the goal of P2–S2 is to reclassify all the samples that were previously predicted as Fusion. Hence, by selecting the feature sets that provided the greatest balanced accuracy and general accuracy in P2–S1, it was possible to focus on the test sets that include more samples from the minority class in the next step.

From the same training sets partitioned in P2–S1, the fusion patients were relabeled as their original classes, HL and OC, to be included in the model development of P2–S2. Besides re-labelling the patients, the training set underwent an oversampling and undersampling process as in P1. The test sets evaluated in P2–S2 were composed of the observations predicted as Fusion from P2–S1. Accordingly, these sets could include not only the actual Fusion samples that are intended to be reclassified but also the wrongly predicted ones, i.e., OPC and NPC that were classified as Fusion in P2–S1. Thus, in order to guarantee that the classifiers of P2–S2 potentially reclassified patients from any class, the training set fed to the model development contained patients from all the four classes.

### 2.4 Inclusion of clinical features

Three clinical features (sex, age and human papillomavirus (HPV) status) were subsequently added to the model training in both P1 and P2 to assess the impact of clinical predictors. These clinical features were integrated into the model as independent features from the selected radiomic feature sets. Sex and HPV status were included in the feature matrix without any alterations, since they were dichotomous variables. On the other hand, age was normalized by z-score standardization.

### 2.5 Explainability analysis

To gain insight into the decision-making processes of the trained model, Shapley value analysis [21] was applied to study model interpretability on the partition that achieved the highest balanced accuracy (over the 30 partition sets). Shapley value, a popular method in explainability analysis studies, considers the order and combination of the features given to the model and computes its contribution for a certain output [21]. Therefore, it is a valuable method for interpreting a model's decision.

First, the Shapley value was computed for each correctly predicted patient of a certain class and provided insight into the relative importance of each feature in the prediction made by the model. Then, after obtaining the Shapley values for each patient, a weighted Shapley value was calculated for each feature in each class based on the number of positive and negative Shapley values present. Equation [1] outlines the method by which the weighted Shapley value for feature *i*, denoted as *wSV*^*i*^, was calculated:

[1] $$wSV^{i}=\left(\frac{{count}^{i,+}}{n}\right)*\;\sum_{{count}^{i,+}}{SV}^{i,+}+\left(\frac{{count}^{i,-}}{n}\right)*\sum_{{count}^{i,-}}{SV}^{i,-}$$

where ${count}^{i,+}$ is the number of patients that were assigned a positive Shapley value for feature *i*; $\sum_{{count}^{i,+}}{SV}^{i,+}$ is the sum of all positive Shapley values of feature *i* obtained from patients in a certain class; ${count}^{i,-}$ and $\sum_{{count}^{i,-}}{SV}^{i,-}$ are analogous to ${count}^{i,+}$ and $\sum_{{count}^{i,+}}{SV}^{i,+}$, respectively; and *n* is the total number of patients in the class being analyzed. This equation can be utilized to determine the weighted Shapley value for any feature under examination.

## 3 Results

A systematic analysis was performed to study the influence of: (i) the machine learning algorithms (NN, NB, DT, BT and SVM), (ii) correlation thresholds (0.75, 0.80 and 0.85) and (iii) feature selection methods (SFS, SBS, SFFS, NCA and MRMR). The comparison of the pipelines proposed (P1 and P2), including the performance of models trained with predictors from different natures (radiomic and clinical), and the analysis of the model explainability were also performed in this study.

### 3.1 Influence of the models

**Fig. 3:**
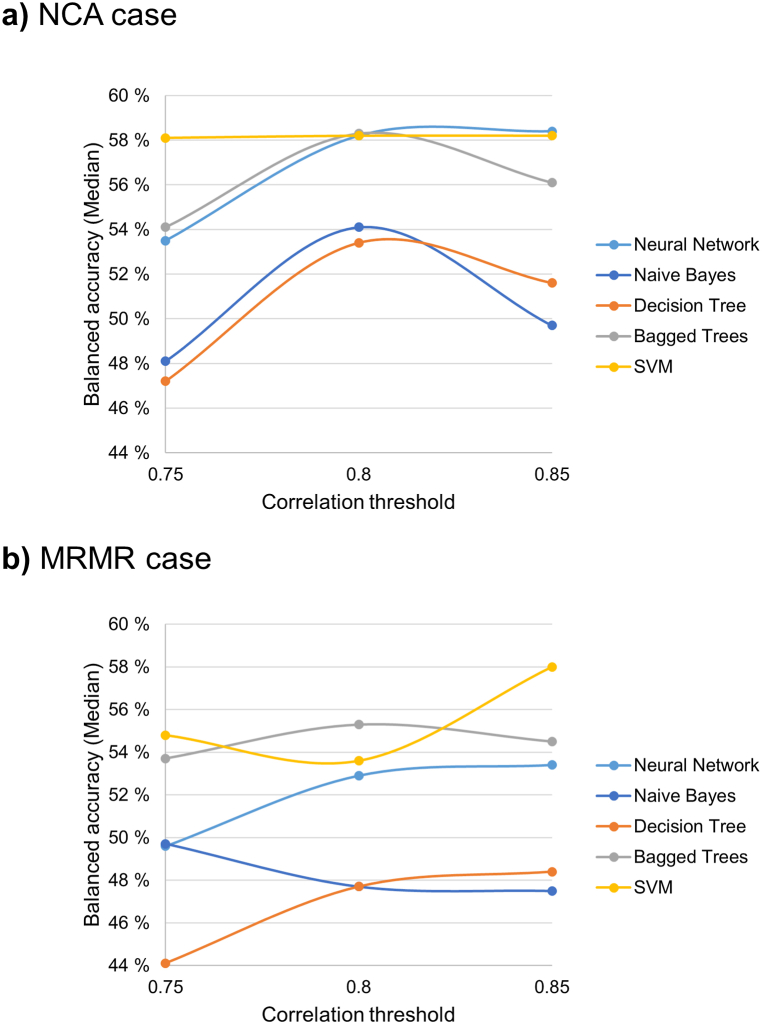
Comparison between the balanced accuracies obtained from different models trained by applying Pipeline 1. The radiomic features included in the model training were selected by **a)** NCA and **b)** MRMR.

The performance of the five models (namely, NN, DT, NB, BT and SVM) in terms of balanced accuracy at different correlation thresholds is shown in Fig. 3, with respect to the feature sets selected by NCA (Fig. 3a) and MRMR methods (Fig. 3b). With the NCA feature selection (Fig. 3a), SVM presented a relatively stable behavior over the three correlation thresholds (0.75, 0.80 and 0.85) outperforming the other models, except for NN which presented similar balanced accuracy for the correlation threshold 0.85. Similarly, also with the MRMR feature selection (Fig. 3b), SVM outperformed the other models except for when the correlation threshold was 0.8. in which case, BT achieved a higher balanced accuracy than SVM. Considering that SVM model achieved the best performances in most the cases among different machine learning models, it was the elected as the best model to evaluate the influence of the correlation thresholds, feature selection methods and pipelines.

### 3.2 Influence of the correlation threshold and feature selection

**Fig. 4:**
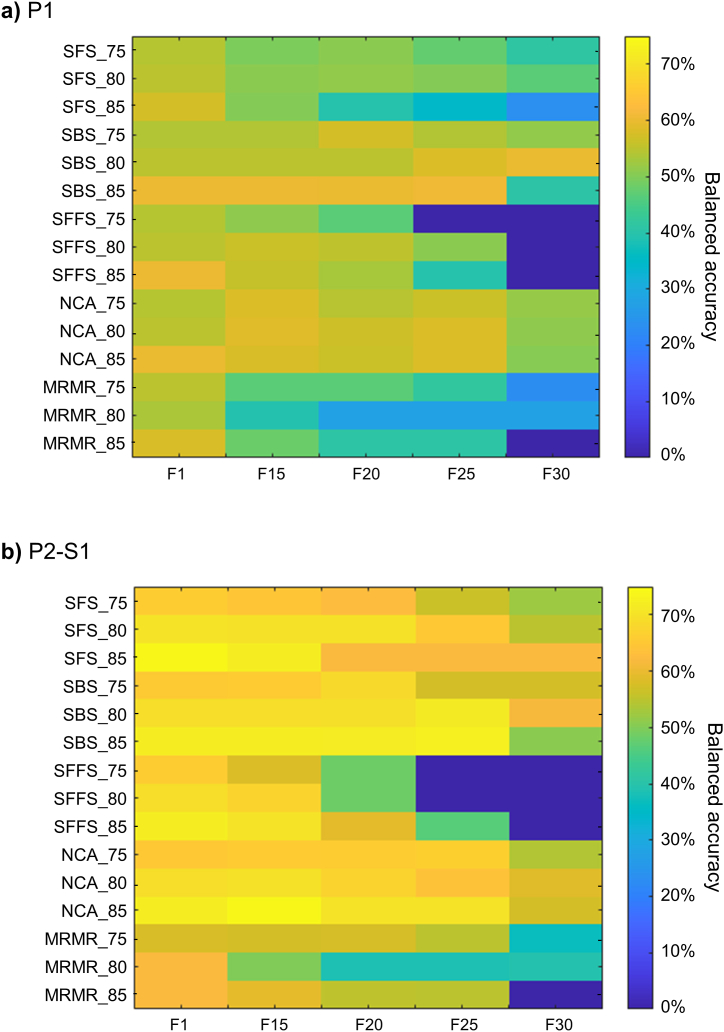
Influence of the feature selection refinement for each feature selection method and correlation threshold in the balanced accuracy of **a)** Pipeline 1 and **b)** Step 1 of Pipeline 2.

The influence of the correlation threshold combined with feature selection method and frequency thresholds on the balanced accuracy is represented in Fig. 4. Each row represents a pair of feature selection method and correlation threshold, and each column shows a frequency threshold. The warm colors in the color map represents higher balanced accuracies in opposite to the cold colors which are associated to lower balanced accuracies. Overall, it is evident that SBS method and the frequency threshold F1 outperformed the other feature selection methods and thresholds both in P1 (Fig. 4a) and in P2–S1 (Fig. 4b). Moreover, the correlation threshold 0.85 was associated with greater balanced accuracies, thus being identified as the optimal threshold.

### 3.3 Influence of the pipelines

The models achieving the best performance in terms of balanced accuracy from each pipeline, either by considering only radiomic or clinical features or their combination, are discussed herein, and the list of radiomic features are presented in Table 2. The accuracies values (%) obtained from those models are summarized in Table 3 as median values along with the first and third quantiles, computed from the results of 30 test and training sets (obtained from the 30-times partition). The balanced accuracy and general accuracy referred to the test set, whereas the training accuracy was obtained from the trained model. Accuracies for individual classes were computed from the sum of the confusion matrices (30 in total, one for each test set partition). Additional results obtained from each pipeline applying different correlation thresholds and feature selection methods can be found in Supplementary Material.

**Table 2:** List of features included in the model construction to achieve the best results.

Features/Pipeline	P1	P2	
		P2S1	P2S2
N_T1w_original_shape_Elongation	x	x	
N_T1w_original_shape_MajorAxisLength	x	x	
N_T1w_original_firstorder_Median	x	x	x
N_T1w_waveletLLH_firstorder_Entropy		x	
N_T1w_waveletLLH_firstorder_Median			x
N_T1w_waveletLLH_glcm_JointEntropy	x	x	
N_T1w_waveletLHL_firstorder_10Percentile	x	x	x
N_T1w_waveletLHL_firstorder_90Percentile		x	
N_T1w_waveletLHL_firstorder_Entropy	x		
N_T1w_waveletLHL_firstorder_Median	x	x	x
N_T1w_waveletLHL_glcm_ClusterProminence	x	x	
N_T1w_waveletLHL_glcm_SumEntropy	x	x	
N_T1w_waveletHLL_glcm_DifferenceEntropy	x	x	
N_T1w_waveletHHH_glcm_ClusterProminence		x	
N_T1w_waveletLLL_firstorder_RobustMean_			
_AbsoluteDeviation		x	
N_T1w_waveletLLL_glcm_DifferenceEntropy	x		
N_T2w_original_shape_Flatness	x	x	
N_T2w_original_shape_LeastAxisLength	x	x	
N_T2w_original_firstorder_Entropy	x	x	
N_T2w_original_firstorder_RootMeanSquared	x	x	
N_T2w_original_firstorder_Skewness	x		x
N_T2w_original_firstorder_TotalEnergy	x	x	
N_T2w_original_glcm_Autocorrelation	x	x	x
N_T2w_original_glcm_Idm	x	x	x
N_T2w_original_glrlm_GrayLevelVariance		x	
N_T2w_waveletLLH_firstorder_Mean	x	x	
N_T2w_waveletLLH_firstorder_Median			x
N_T2w_waveletLLH_glcm_ClusterProminence	x	x	
N_T2w_waveletLLH_glcm_MaximumProbability	x		x
N_T2w_waveletLHL_glcm_DifferenceEntropy	x	x	
N_T2w_waveletLHL_glcm_SumEntropy	x	x	
N_T2w_waveletHHL_glrlm_			
_GrayLevelNonUniformityNormalized		x	
N_T2w_waveletHHH_firstorder_Entropy	x	x	x
N_T2w_waveletLLL_firstorder_InterquartileRange	x	x	
N_T2w_waveletLLL_firstorder_Range	x	x	x
N_T2w_waveletLLL_firstorder_Skewness		x	
N_T2w_waveletLLL_glcm_Autocorrelation	x		
N_T2w_waveletLLL_glcm_MaximumProbability			x
Total no. radiomic features	28	30	12
Correlation threshold	0,85	0,85	0,80
Feature selection method	SBS	NCA	MRMR
Histogram threshold	F25	F15	F1

P1 = pipeline 1; P2S1 = first step of pipeline 2; P2S2 = second step of pipeline 2; SBS = sequential backwards selection; NCA = neighborhood component analysis; MRMR = minimum redundancy maximum relevance.

**Table 3:** Summary of results obtained from each pipeline trained with radiomic features, with clinical features, and with radiomics plus clinical features. The results presented are the best ones obtained from each pipeline, based on the balanced accuracy and general accuracy. The accuracies for each class were computed from the sum of the confusion matrices (30 in total, one for each test set partition). The models trained in Pipeline 1 are quadratic support vector machine (SVM) and in Pipeline 2 linear SVM.

	Median [IQR]			Accuracy			
	Bal_acc (%)	Gen_acc (%)	Train_acc (%)	HL(%)	OC(%)	OPC(%)	NPC(%)
**Radiomic features**							
P1	60.7 [52.9; 68.3]	65.0 [62.5; 70.0]	68.6 [66.7; 70.2]	45.0	52.8	51.7	91.9
P2–S1	74.0 [67.7; 77.9]	77.5 [72.5; 82.5]	75,0 [74.2; 75,8]	–	–	88.5	93.8
P2–S2	47.2 [33.3; 66.7]	50.0 [33.3; 66.7]	58,5 [55.9; 59.5]	66.7	78.6	–	–
**Clinical features**							
P1	64.8 [54.9; 69.7]	62.5 [55.0; 70.0]	63.2 [62.3; 65.4]	70.0	62.2	64.8	56.0
P2–S1	74.7 [70.5; 80.1]	72.5 [67.5; 77.5]	71.4 [70.6; 72.2]	–	–	62.7	61.0
P2–S2	33.4 [28.1; 40.0]	34.3 [28.6; 40.0]	61.4 [59.7; 63.2]	77.2	44.2	–	–
**Radiomic + Clinical features**							
P1	69.4 [66.2; 79.2]	76.3 [70.0; 82.5]	79.4 [78.5; 81.1]	50.8	73.3	71.5	89.5
P2–S1	88.5 [83.6; 91.7]	87.5 [82.5; 90.0]	86.7 [86.4; 86.9]	–	–	71.3	92.9
P2–S2	48.3 [37.5; 55.6]	50.0 [42.9; 57.1]	74.8 [73.7; 76.3]	75.3	75.4	–	–

P1 = pipeline 1; P2–S1 = first step of pipeline 2; P2–S2 = second step of pipeline 2; Bal_acc = balanced accuracy; Gen_acc = general accuracy; Train_acc = training accuracy; HL = hypopharynx and larynx; OC = oral cavity; OPC = oropharynx; NPC = nasopharynx.

The analysis of the influence of the pipelines and the models (radiomic vs clinical vs radiomic-clinical) was based on the balanced accuracy, being the most valuable performance metric for our multiclassification problem, and the individual accuracies of the classes. In terms of balanced accuracy, the results for P1 showed that the combined radiomic-clinical model outperformed the radiomic model and clinical model alone, i.e., balanced accuracy: 69.4 % (radiomic + clinical) vs 60.7 % (radiomic) vs 64.8 % (clinical). The same was observed for P2, where the radiomic-clinical model outperformed the other ones, i.e., balanced accuracy: 81.9 % (radiomic + clinical) vs 78.7 % (radiomic) vs 61.3 % (clinical). As regards the influence of the pipelines (P1 vs P2), the P2-radiomic model outperformed the P1-radiomic model in classifying all the class (HL: 45.0 % (P1) vs 66.7 % (P2); OC: 52.8 % (P1) vs 78.6 % (P2); OPC: 51.7 %(P1) vs 88.5 % (P2); NPC: 91.9 %(P1) vs 93.8 % (P2)). On the other hand, the P1-clinical model outperformed the P2-clinical model in classifying the classes OC and OPC (OC: 62.2 % (P1) vs 44.2 % (P2); OPC: 64.8 % (P1) vs 62.7 % (P2)) while P2 achieved a higher accuracy for the classes HL and NPC (HL: 70.0 % (P1) vs 77.2 % (P2); NPC: 56.0 % (P1) vs 61.0 % (P2)). Concerning the radiomic-clinical models, P2 outperformed P1, achieving a better accuracy for every class except for OPC which obtained a similar accuracy for both pipelines (HL: 50.8.0 % (P1) vs 75.3 % (P2); OC: 73.3 % (P1) vs 75.4 % (P2); OPC: 71.5 % (P1) vs 71.3 % (P2); NPC: 89.5 % (P1) vs 92.9 % (P2)).

### 3.4 Explainability analysis

Table 4, Table 5 synthesizes the features with the greatest impact (both positive and negative) on the model's prediction for each class (OPC, NPC, OC and HL), based on the weighted Shapley value. Fusion was not included in these summary tables since it is only a transitory class.

**Table 4:** Summary of the most positive impactful features and its weighted Shapley values for each class. Positive impact features are the ones that obtained a positive weighted Shapley value.

Class	Positive Impact Features	Weighted Shapley value
**OPC**	N_T2w_waveletLHL_glcm_DifferenceEntropy	0.0253
**n = 15/16**	N_T1w_waveletLHL_firstorder_10Percentile	0.0233
**acc = 93.8 %**	N_T1w_waveletLHL_firstorder_90Percentile	0.0220

**NPC**	HPV_status	0.0849
**n = 13/14**	N_T2w_waveletHHH_firstorder_Entropy	0.0584
**acc = 92.9 %**	Age	0.0228

**OC**	N_T1w_waveletLHL_glcm_SumEntropy	0.0050
**n = 5/7**	N_T1w_original_firstorder_Median	0.0013
**acc = 71.4 %**	N_T1w_original_shape_Elongation	0.0010

**HL**	N_T1w_original_shape_Elongation	0.0173
**n = 3/3**	N_T1w_waveletLHL_glcm_SumEntropy	0.0086
**acc = 100 %**	N_T1w_waveletLLH_firstorder_Entropy	0.0054

n: number of patients correctly classified by the model in analysis divided by the total number of existent patients from the test set in evaluation; acc: accuracy of the class.

**Table 5:** Summary of the most negative impactful features and its weighted Shapley values for each class. Negative impact features are the ones that obtained a positive weighted Shapley value.

Class	Negative Impact Feature	Weighted Shapley value
**OPC**	N_T2w_waveletLHL_glcm_SumEntropy	−0.0522
**n = 15/16**	N_T1w_original_shape_MajorAxisLength	−0.0256
**acc = 93.8 %**	N_T2w_original_firstorder_Entropy	−0.0246

**NPC**	N_T1w_original_shape_MajorAxisLength	−0.0492
**n = 13/14**	N_T1w_waveletLHL_glcm_ClusterProminence	−0.0312
**acc = 92.9 %**	N_T1w_waveletLLH_firstorder_Entropy	−0.0205

**OC**	N_T1w_waveletLHL_firstorder_Median	−0.0109
**n = 5/7**	N_T1w_original_shape_MajorAxisLength	−0.0048
**acc = 71.4 %**	N_T2w_original_shape_LeastAxisLength	−0.0042

**HL**	N_T1w_original_firstorder_Median	−0.0082
**n = 3/3**	N_T1w_waveletLLL_firstorder_RobustMeanAbsoluteDeviation	−0.0072
**acc = 100 %**	N_T1w_waveletHLL_glcm_DifferenceEntropy	−0.0046

n: number of patients correctly classified by the model in analysis divided by the total number of existent patients from the test set in evaluation; acc: accuracy of the class.

*HPV_status* was the feature with the highest positive impact with a weighted Shapley value of 0.0849, followed by *N_T2w_waveletHHH*_firstorder_Entropy with a value of 0.0584. On the other hand, *N_T2w_waveletLHL_glcm_SumEntropy* was the predictor with the highest negative impact with a weighted Shapley value of −0.0522, followed by *N_T1w_original_shape_MajorAxisLength* with a value of −0.0492.

Furthermore, the Shapley analysis allowed identifying the most impactful predictors of each primary tumor locations under study. For the OPC class, the N_T2w_waveletLHL_glcm_SumEntropy predictor, linked to texture analysis through the Grey-Level Co-occurrence Matrix (GLCM), was the most impactful variable. GLCM provides information on the texture and patters of a ROI by quantifying the frequency of pixel value pairing in relation to spatial relationships. The NPC class was strongly influenced by the HPV_status predictor, consistent with clinical findings [22]. OC classification relied mostly on the N_T1w_waveletLHL_firstorder_Median predictor, a statistic-derived pixel distribution feature, specifically median values. In the HL class, the N_T1w_original_shape_Elongation predictor, belonging to the shape-size class, assesses tumor morphology by quantifying deviation from spherical shape, was found to be the most impactful variable.

## 4 Discussion

In the present study two radiomic-based machine learning multiclassification pipelines were developed based on a dataset composed of HNC patients to predict the primary tumor site in patients with head and neck carcinoma. Specifically, P1 consisted in a direct identification of the primary sites, whereas the second pipeline was based on a two-step approach: in the first step, the number of classes was reduced by merging HL with OC, which were then reclassified in the second step. Both pipelines included the following four stages: (i) Feature preprocessing, (ii) Data partition, (iii) Feature selection, (iv) Classification model development. The results demonstrated the higher performance of the developed two-step multiclassification pipeline (P2) in addressing the multiclassification problem.

In both pipelines, radiomic, clinical and radiomic-clinical models were developed, where the clinical features included sex, age and virus information. Results of our analysis suggested that models trained with radiomic features in combination with clinical information are the most promising in increasing the model's accuracy.

Furthermore, a systematic analysis was performed in which, the influence of (i) multiple machine learning algorithms (NN, NB, DT, BT and SVM), (ii) correlation thresholds (0.75, 0.80 and 0.85) and (iii) feature selection methods (SFS, SBS, SFFS, NCA and MRMR) were assessed. Specifically, SVM outperformed NN, NB, DT and BT, and the correlation threshold of 0.85 and the SBS feature selection method resulted in larger balanced accuracies, indicating that they are the most effective.

Finally, an explainability analysis was conducted in order to gain insight in the trained model. In particular, the analysis revealed that the most impactful feature to classify the OPC class was *N_T2w_waveletLHL_glcm_SumEntropy*; the NPC class was *HPV_status*; the OC class was *N_T1w_waveletLHL_firstorder_Median* and the HL class was *N_T1w_original_shape_Elongation*.

To the best of the authors’ knowledge, radiomic-based multiclassification has never been applied to address this clinical issue, i.e., predict the location of the primary tumor in HNC patients. Our results are in line with the only previous study dealing with the same problem [10], employing DNA methylation profiles to develop machine learning models that achieved high classification accuracies, ranging from 83 % to 89 %. While their approach shows promise, it involves the invasive and less ideal procedure of obtaining a cervical lymph node biopsy to perform the DNA methylation analysis. In contrast, our developed machine learning pipelines are non-invasive and utilize radiomic features extracted from routine imaging, making them more patient-friendly and accessible.

Furthermore, according to the authors' understanding, few studies have applied radiomics to predict unknown primary tumors sites, and the existent ones mostly lack in exploring and analyzing the methodological aspects [[7], [8], [9]]. Additionally, the existent studies have focused on distinguishing metastases from primary tumors located in distant regions of the body, whereas our investigation sought to predict the location of primary tumors within the same anatomical region, specifically the head and neck region. For example, Ortiz-Ramón et al. [7] built three-class radiomic models to differentiate brain metastases from lung cancer, breast cancer and melanoma, but they only assessed one feature selection method based on the p-value and trained in one machine learning model (random forest). Their results were satisfactory in classifying lung cancer brain metastasis (acc = 82 %) but unsatisfactory in classifying breast cancer (acc = 58.8 %) and melanoma brain metastases (acc = 67.8 %). Similarly, Kniep et al. [9] built radiomic models to predict brain metastasis assessing only one feature selection method based on Gini impurity measures and only on the random forest model. However, they included two more primary cancers and built both three-class (small cell lung cancer, breast cancer and melanoma) and five-class models (small cell lung cancer, breast cancer, melanoma, gastrointestinal cancer and non-small cell lung cancer). As expected, the three-class model achieved better results than the five-class model, obtaining a similar range of sensitivity and specificity of 76 %–80 % for the three-class model and a range of sensitivity and specificity of 58 %–74 % and 58 %–76 %, respectively, for the 5-class model. Moreover, Kniep et al. [9] faced an imbalance dataset problem and addressed it in adjusting the class weights during the training phase but no change was observed in the results. None of the mentioned methods compared feature selection methods nor classifier models, and their methodology were typically similar to the P1 but without addressing imbalanced dataset problem properly. Our study focused on evaluating diverse feature selection methods and machine learning models, while addressing the imbalanced dataset problem using over- and undersampling techniques. In addition, our methodology minimized the results bias due to the 30 times partition of the dataset in the *data partition* step, allowing the computation of a median accuracy and the respective quantiles. Between the two pipelines we proposed for the multiclassification challenge, the best results were achieved by the second pipeline when clinical information was included in the feature set selected by sequential backward selection and trained with a linear SVM classifier. The highest accuracies obtained in predicting each tumor location were 75.3 % for HL, 75.4 % for OC, 71.3 % for OPC and 92.9 % for NPC. Despite our efforts to address the class imbalance, we observed that the classification results were not highly homogeneous across all classes, with NPC presenting noticeably higher accuracy compared to the other classes. In addition, the results support the value of our methodology, aligning with Hosseinzadeh et al.'s [23] emphasis on combining diverse features, including clinical and radiomics, and advanced techniques result in best prediction performance.

The present study is not exempt from limitations. For instance, although a selection of models was evaluated, other models like the random forest algorithm which was proven to be efficient in Kniep et al. [9] and Ortiz-Ramón et al.’s [7] work, was not considered herein. Deep learning algorithm has also been shown in the literature to be a powerful method in multiclassification challenges and in predicting treatment outcomes and survival [[24], [25], [26]]. Thus, future work could be invested in exploring the random forest algorithm as well as deep learning methods. Given that our study is based on data from a single center, it's important to acknowledge that this could affect the generalizability of the findings. Therefore, future endeavors should also focus on validating the developed models using external datasets, and subsequently conducting clinical trials to assess their real-world impact. Despite the limitations identified, our work demonstrated robustness in comparing not only extensively different machine learning algorithms but also feature selection methods and correlation thresholds.

In conclusion, the main contribution of our work is the development of a radiomic model that has proven to be a valuable tool for clinical decision support in predicting the primary tumor site of head and neck carcinoma of unknown primary. It guides clinicians to the most probable primary tumor site, reducing the need for multiple examinations and, as a result, significantly improving current diagnostic methods. Between the two radiomic-based machine learning multiclassification pipeline developed in this study, the two-step multiclassification pipeline, P2, demonstrated higher performance in predicting the primary tumor site of head and neck carcinoma of unknown primary based on a dataset composed of HNC patients. Both pipelines were applied for the first time in this HNC dataset to predict the primary tumor site of head and neck carcinoma with unknown primary, based on radiomic features. The satisfactory results obtained in identifying this highly heterogeneous disease makes it a promising approach for other tumor metastases with unknown primary.

## Ethics statement

Patients included in this study were part of the BD2Decide project (ClinicalTrial.gov Identifier: NCT02832102), a multi-centric clinical study in locally advanced HNC [11]. The conduction of these studies was approved by Institutional Ethical Committee of Istituto Nazionale dei Tumori, Milan, Italy (study protocol identifiers INT65-16 and INT66-16 for the BD2Decide)**,** and data acquisition followed the General Data Protection Regulation of the EU.

## Data availability

The datasets used and analyzed during the current study are available from the corresponding author on reasonable request. All relevant materials have been included in the article and its supplementary data files.

## CRediT authorship contribution statement

**Jiaying Liu:** Writing – review & editing, Writing – original draft, Methodology, Formal analysis. **Anna Corti:** Writing – review & editing, Visualization, Investigation, Formal analysis. **Giuseppina Calareso:** Writing – review & editing, Data curation. **Gaia Spadarella:** Writing – review & editing, Conceptualization. **Lisa Licitra:** Writing – review & editing, Data curation. **Valentina Corino:** Writing – review & editing, Visualization, Supervision, Investigation, Formal analysis, Conceptualization. **Luca Mainardi:** Writing – review & editing, Visualization, Supervision, Investigation, Formal analysis, Conceptualization.

## Declaration of competing interest

The authors declare that they have no known competing financial interests or personal relationships that could have appeared to influence the work reported in this paper.
