# Supplementary material for: Developing a robust two-step machine learning multiclassification pipeline to predict primary site in head and neck carcinoma from lymph nodes
Source: Heliyon. 2024 Jan 12;10(2):e24377. doi: 10.1016/j.heliyon.2024.e24377 (PMC10835257; doi:10.1016/j.heliyon.2024.e24377)
Supplement: Multimedia component 1 [file mmc1.docx]

**Developing a robust two-step machine learning multiclassification pipeline to predict primary site in head and neck carcinoma from lymph nodes**

**Supplementary Information**

Jiaying Liu^1,*^, Anna Corti^2^, Giuseppina Calareso^3^, Gaia Spadarella^4,5^, Lisa Licitra^6,7^, Valentina Corino^1,8^, Luca Mainardi^1^

1. Department of Electronics, Information and Bioengineering, Politecnico di Milano, Milan, Italy
2. Laboratory of Biological Structure Mechanics (LaBS), Department of Chemistry, Materials and Chemical Engineering “Giulio Natta”, Politecnico di Milano, Milan, Italy
3. Radiology Department, Fondazione IRCCS Istituto Nazionale dei Tumori di Milano, Milan, Italy
4. Postgraduation School in Radiodiagnostics, University of Milan, Italy
5. Department of Clinical Medicine and Surgery, Federico II University, Naples, Italy
6. Head and Neck Cancer Medical Oncology 3 Department, Fondazione IRCCS Instituto Nazionale dei Tumori di Milano, Milan, Italy
7. Department of Oncology and Hemato-Oncology, University of Milan, Italy
8. Cardiotech Lab, Centro Cardiologico Monzino IRCCS, Milan, Italy

***Address for correspondence:**

Jiaying Liu

Department of Electronics, Information and Bioengineering, Politecnico di Milano, Milan, Italy

[jiaying.liu@polimi.it](mailto:jiaying.liu@polimi.it)

1. **Feature selection methods**

Sequential forward selection (SFS) is a method that iteratively selects features by adding one feature at a time, evaluating their contribution to the model's performance. It starts with an empty set and adds features that maximize the classifier performance.

Sequential backwards selection (SBS) is the reverse of SFS, starting with all available features and removing them one by one to assess their impact on the model's performance.

Sequential forward floating selection (SFFS) is a variation of SFS that allows for features to be added and removed during the selection process, potentially improving the final feature set's quality.

The neighbourhood component analysis (NCA) is a non-parametric method for selecting features with the goal of maximizing prediction accuracy of regression and classification algorithms.

Minimum redundancy maximum relevance (MRMR) algorithm is designed to select features based on their relevance to the target variable and their redundancy with other features. It seeks a balance between these two aspects to identify an informative and non-redundant feature set.

Both SFS and SBS were applied in the study using Matlab function *sequentialfs*. NCA and MRMR algorithms were assessed using Matlab function *fscnca* and *fscmrmr*.

SFFS method was implemented in Matlab following the steps described in the flow chart in Figure S1. It is possible to identify two steps: one that includes features and a second one that removes features.


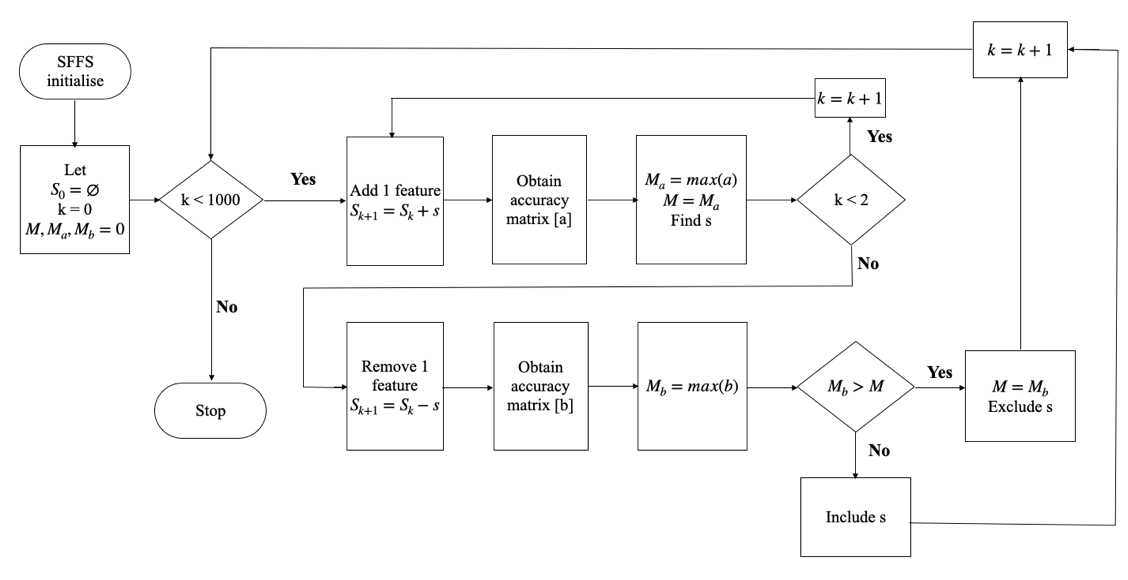


**Figure S1:** Flow chart of SFFS algorithm. The algorithm starts with an empty set of features ($S_{0}$) and adds the feature $s$ that gives the best prediction accuracy from a model trained. $M_{a}$ is the maximum accuracy obtained on the forward step. After each forward step, SFFS performs a backward step and selects the maximum accuracy ($M_{b}$) computed by removing one feature. These two maximums are compared, the highest accuracy is selected, and the correspondent feature set follows to the next iteration. The algorithm stops when $k$ = 1000.

The algorithm initialises an empty feature set ($S_{0}$), a maximum accuracy ($M$) and an iteration count ($k$) set as zero. It starts by adding one feature s to the feature set and trains as many SVM models using fitcecoc as the number of existing features. While models are trained and the accuracy is assessed, a matrix with the size of the number of features is filled in with the values obtained. Then the feature that achieves the best accuracy is kept in the set. The second process of this algorithm consists of excluding feature by feature after choosing the best features in the first process. Again, the accuracy is evaluated and compared with previously stored maximum accuracy. If the accuracy improves by excluding a feature $s$, then the feature is removed. Accordingly, if by excluding any of the features, the accuracy does not improve, then the feature set remains the same. This method becomes infeasible when we want to assess every possible combination since it is extremely time-consuming. Thus, the number of iterations was limited to stop when $k$ = 1000. This way, it is possible to evaluate the different combinations dynamically and find the optimal feature set.

1. **Radiomic features**

Table S1 lists the radiomic features present in the feature matrix after removing the highly correlated ones in step 1. *feature preprocessing,* performed in the same way for P1 and P2.

**Table S1:** List of features remained in the feature matrix after step 1. *feature preprocessing*. Different correlation thresholds (0.75, 0.80, 0.85) are separated by columns

| **Features/Thresholds** | **0.85** | **0.80** | **0.75** |
| --- | --- | --- | --- |
| N_T1w_original_shape_Elongation | x |  |  |
| N_T1w_original_shape_MajorAxisLength | x | x | x |
| N_T1w_original_firstorder_Median | x | x | x |
| N_T1w_original_firstorder_TotalEnergy | x | x | x |
| N_T1w_original_glcm_InverseVariance | x | x |  |
| N_T1w_waveletLLH_firstorder_Entropy | x | x |  |
| N_T1w_waveletLLH_firstorder_Mean | x | x | x |
| N_T1w_waveletLLH_firstorder_Median | x | x | x |
| N_T1w_waveletLLH_glcm_JointEntropy | x | x | x |
| N_T1w_waveletLHL_firstorder_10Percentile | x | x | x |
| N_T1w_waveletLHL_firstorder_90Percentile | x |  |  |
| N_T1w_waveletLHL_firstorder_Entropy | x |  |  |
| N_T1w_waveletLHL_firstorder_Median | x | x | x |
| N_T1w_waveletLHL_glcm_ClusterProminence | x |  |  |
| N_T1w_waveletLHL_glcm_SumEntropy | x | x | x |
| N_T1w_waveletHLL_firstorder_Median | x | x | x |
| N_T1w_waveletHLL_glcm_DifferenceEntropy | x | x | x |
| N_T1w_waveletHHH_glcm_ClusterProminence | x |  |  |
| N_T1w_waveletLLL_firstorder_  _RobustMeanAbsoluteDeviation | x | x | x |
| N_T1w_waveletLLL_glcm_DifferenceEntropy | x |  |  |
| N_T2w_original_shape_Flatness | x | x | x |
| N_T2w_original_shape_LeastAxisLength | x |  |  |
| N_T2w_original_firstorder_Entropy | x | x | x |
| N_T2w_original_firstorder_RootMeanSquared | x | x | x |
| N_T2w_original_firstorder_Skewness | x | x | x |
| N_T2w_original_firstorder_TotalEnergy | x |  |  |
| N_T2w_original_glcm_Autocorrelation | x | x |  |
| N_T2w_original_glcm_Idm | x | x |  |
| N_T2w_original_glrlm_GrayLevelVariance | x | x | x |
| N_T2w_waveletLLH_firstorder_Mean | x |  |  |
| N_T2w_waveletLLH_firstorder_Median | x | x | x |
| N_T2w_waveletLLH_glcm_ClusterProminence | x | x | x |
| N_T2w_waveletLLH_glcm_MaximumProbability | x | x | x |
| N_T2w_waveletLHL_glcm_DifferenceEntropy | x | x |  |
| N_T2w_waveletLHL_glcm_SumEntropy | x | x | x |
| N_T2w_waveletHHL_glrlm_  _GrayLevelNonUniformityNormalized | x | x | x |
| N_T2w_waveletHHH_firstorder_Entropy | x | x | x |
| N_T2w_waveletLLL_firstorder_Entropy | x | x |  |
| N_T2w_waveletLLL_firstorder_InterquartileRange | x | x |  |
| N_T2w_waveletLLL_firstorder_Range | x | x |  |
| N_T2w_waveletLLL_firstorder_Skewness | x |  |  |
| N_T2w_waveletLLL_glcm_Autocorrelation | x |  |  |
| N_T2w_waveletLLL_glcm_MaximumProbability | x | x | x |
| **Total** | 43 | 32 | 24 |

1. **Other results obtained from P1 and P2**

**P1: Radiomic model performance**

**Table S2**: Results obtained from P1 using radiomic features and training Quadratic SVM.

| **Feat. Selection** | **Bal_acc(%)** | | **Gen_acc(%)** | | **Train_acc(%)** | | **#Feat** | | |
| --- | --- | --- | --- | --- | --- | --- | --- | --- | --- |
| **Thr = 0.75** |  |  | |  | |  | | |  |
| - | 54.4 | | 60.0 | | 64.7 | | 24 | - | |
| SFS | 50.7 | | 55.0 | | 57.9 | | 8 | F20 | |
| SBS | 53.9 | | 67.5 | | 65.8 | | 6 | F30 | |
| SFFS | 51.2 | | 57.5 | | 61.6 | | 15 | F15 | |
| NCA | 56.5 | | 61.3 | | 65.1 | | 12 | F25 | |
| MRMR | 54.8 | | 60.0 | | 63.6 | | 19 | F1 | |
| **Thr = 0.80** |  | |  | |  | |  |  | |
| - | 55.0 | | 62.5 | | 67.1 | | 32 | - | |
| SFS | 51.5 | | 56.3 | | 52.9 | | 4 | F20 | |
| SBS | 60.0 | | 65.0 | | 62.5 | | 7 | F30 | |
| SFFS | 55.1 | | 54.8 | | 61.2 | | 13 | F20 | |
| NCA | 58.2 | | 65.0 | | 59.0 | | 5 | F25 | |
| MRMR | 53.6 | | 57.5 | | 62.9 | | 23 | F1 | |
| **Thr = 0.85** |  | |  | |  | |  |  | |
| - | 60.3 | | 65.0 | | 67.8 | | 43 | - | |
| SFS | 57.3 | | 62.5 | | 67.5 | | 40 | F1 | |
| SBS | 60.7 | | 65.0 | | 68.6 | | 28 | F25 | |
| SFFS | 55.8 | | 61.3 | | 66.0 | | 28 | F15 | |
| NCA | 58.2 | | 68.3 | | 60.1 | | 7 | F25 | |
| MRMR | 58.0 | | 62.5 | | 65.4 | | 30 | F1 | |

Feat. = feature; Thr = correlation threshold; Bal_acc = balanced accuracy; Gen_acc =

general accuracy; Train_acc = training accuracy; #Feat = number of features used and

correspondent frequency threshold.

**P1: Clinical model performance**

**Table S3**: Results obtained from P1 using clinical features and training a Quadratic SVM.

| **Clinical features** | **Bal_acc(%)** | **Gen_acc(%)** | **Train_acc(%)** | **#Feat** |
| --- | --- | --- | --- | --- |
| Sex, Age, HPV | 64.8 | 62.5 | 63.2 | 3 |
| Bal_acc = balanced accuracy; Gen_acc = general accuracy; Train_acc = training accuracy; #Feat = number of features used. | | | | |

**P1: Radiomic-clinical model performance**

**Table S4**: Results obtained from P1 using radiomic and clinical features, and training Quadratic SVM.

| **Feat. Selection** | **Bal_acc(%)** | | **Gen_acc(%)** | | **Train_acc(%)** | | **#Feat** | | |
| --- | --- | --- | --- | --- | --- | --- | --- | --- | --- |
| **Thr = 0.75** |  |  | |  | |  | | |  |
| - | 71.8 | | 73.8 | | 78.7 | | 24 + 3 | - | |
| SFS | 72.4 | | 73.8 | | 78.1 | | 8 + 3 | F20 | |
| SBS | 73.7 | | 75.0 | | 78.5 | | 20 + 3 | F20 | |
| SFFS | 69.9 | | 72.5 | | 76.5 | | 15 + 3 | F15 | |
| NCA | 75.7 | | 72.5 | | 78.1 | | 12 + 3 | F25 | |
| MRMR | 70.9 | | 75.0 | | 78.1 | | 19 + 3 | F1 | |
| **Thr = 0.80** |  | |  | |  | |  |  | |
| - | 71.5 | | 75.0 | | 78.7 | | 32 + 3 | - | |
| SFS | 68.3 | | 72.5 | | 75.4 | | 7 + 3 | F15 | |
| SBS | 73.1 | | 75.0 | | 79.0 | | 20 + 3 | F25 | |
| SFFS | 73.3 | | 75.0 | | 79.0 | | 13 + 3 | F20 | |
| NCA | 73.1 | | 77.5 | | 76.3 | | 5 + 3 | F25 | |
| MRMR | 71.9 | | 70.0 | | 77.4 | | 23 + 3 | F1 | |
| **Thr = 0.85** |  | |  | |  | |  |  | |
| - | 73.3 | | 75.0 | | 79.8 | | 43 + 3 | - | |
| SFS | 74.8 | | 77.5 | | 80.7 | | 40 + 3 | F1 | |
| SBS | 73.4 | | 75.0 | | 79.0 | | 39 + 3 | F25 | |
| SFFS | 74.1 | | 76.3 | | 80.0 | | 28 + 3 | F15 | |
| NCA | 72.7 | | 75.0 | | 79.2 | | 12 + 3 | F20 | |
| MRMR | 73.5 | | 73.8 | | 77.9 | | 30 + 3 | F1 | |
| Feat. = feature; Thr = correlation threshold; Bal_acc = balanced accuracy; Gen_acc = general accuracy; Train_acc = training accuracy; #Feat = number of radiomic features used (plus 3 clinical features) and correspondent frequency threshold. | | | | | | | | | |

**Comparing the obtained results from P1**

**Table S5**: Comparison of the results obtained from P1 by applying radiomic features, clinical features and radiomic with clinical features. The values presented for each performance metric (Bal_acc, Gen_acc and Val_acc) are the average of the results presented in Table B.1 and Table B.2, i.e., for each column of the table (corresponding to a performance metric), the average is computed regardless of the correlation threshold. The results obtained by applying only clinical features are the same ones presented in Table B.3 without computing the average.

| **P1** | **Average** | | |
| --- | --- | --- | --- |
|  | **Bal_acc(%)** | **Gen_acc(%)** | **Train_acc(%)** |
| Radiomic model | 55.9 | 61.5 | 63.3 |
| Clinical model | 64.8 | 62.5 | 63.2 |
| Radiomic-clinical model | 72.6 | 74.5 | 78.3 |
| P1 = pipeline 1; Bal_acc = balanced accuracy; Gen_acc = general accuracy; Train_acc = training accuracy. | | | |

**P2**

Since P2 comprises two separate stages of classification, the results are also exposed individually. Therefore, an outcome table for P2-S1 and another for P2-S2 are included in this section. Moreover, a third table containing the results obtained by combining different feature sets in both steps is shown. The feature sets analysed in the third table is selected based on a new metric - mean - computed by averaging accuracies (balance accuracy and individual accuracies) of each feature selection method in the first two tables (one for P2-S1 and another for P2-S2). In both steps, for each correlation threshold, a best feature select is selected, i.e., three feature sets are chosen in the first step and combined with three feature sets chosen in the second step, resulting in nine combinations as shown in Table S8 and S11.

**P2: Radiomic model performance**

**Table S6**: Results obtained from P2-S1 using radiomic features and training Linear SVM.

| **Feat. Selection** | **Bal_acc(%)** | | **OPh_acc (%)** | | **NPh_acc(%)** | | **#Feat** | | | **Mean** |
| --- | --- | --- | --- | --- | --- | --- | --- | --- | --- | --- |
| **Thr = 0.75** |  |  | |  | |  | | |  |  |
| -* | 66.2 | | 83.3 | | 87.1 | | 24 | - | | 87.1 |
| SFS | 64.5 | | 83.3 | | 85.2 | | 9 | F15 | | 77.2 |
| SBS | 68.4 | | 81.9 | | 84.3 | | 15 | F25 | | 78.2 |
| SFFS | 58.1 | | 83.3 | | 83.6 | | 11 | F15 | | 71.0 |
| NCA | 66.2 | | 82.9 | | 85.0 | | 19 | F20 | | 78.0 |
| MRMR | 57.6 | | 82.7 | | 70.2 | | 6 | F20 | | 70.2 |
| **Thr = 0.80** |  | |  | |  | |  |  | |  |
| - | 69.4 | | 87.7 | | 89.5 | | 32 | - | | 82.2 |
| SFS | 70.0 | | 85.4 | | 88.8 | | 5 | F15 | | 81.4 |
| SBS* | 71.5 | | 85.4 | | 90.0 | | 19 | F25 | | 82.3 |
| SFFS | 67.4 | | 79.4 | | 91.7 | | 13 | F15 | | 79.5 |
| NCA | 70.0 | | 83.5 | | 88.8 | | 14 | F15 | | 80.8 |
| MRMR | 62.2 | | 65.0 | | 64.2 | | 12 | F1 | | 63.8 |
| **Thr = 0.85** |  | |  | |  | |  |  | |  |
| - | 72.2 | | 86.3 | | 94.3 | | 43 | - | | 84.2 |
| SFS | 72.1 | | 84.8 | | 94.3 | | 6 | F20 | | 83.7 |
| SBS | 72.0 | | 86.3 | | 94.3 | | 42 | F30 | | 84.2 |
| SFFS | 70.3 | | 84.8 | | 93.6 | | 23 | F15 | | 82.9 |
| NCA* | 74.0 | | 88.8 | | 93.8 | | 30 | F25 | | 85.5 |
| MRMR | 61.9 | | 65.0 | | 65.6 | | 18 | F1 | | 64.2 |
| Feat. = feature; Thr = correlation threshold; Bal_acc = balanced accuracy; OPh_acc = oropharyx accuracy; NPh_acc = nasopharynx accuracy; #Feat = number of features used and correspondent frequency threshold. Mean is the average of Bal_acc, OPh_acc and NPh_acc; * = feature set that obtained the best mean compared to the other feature sets from the same correlation threshold. | | | | | | | | | | |

**Table S7**: Results obtained from P2-S2 using radiomic features and training Linear SVM.

| **Feat. Selection** | **Bal_acc(%)** | | **HL_acc (%)** | | **OC_acc(%)** | | **#Feat** | | | **Mean** |
| --- | --- | --- | --- | --- | --- | --- | --- | --- | --- | --- |
| **Thr = 0.75** |  |  | |  | |  | | |  |  |
| -* | 50.0 | | 59.3 | | 92.7 | | 24 | - | | 67.3 |
| SFS | 50.0 | | 52.0 | | 85.3 | | 9 | F15 | | 62.4 |
| SBS | 50.0 | | 72.5 | | 74.7 | | 15 | F25 | | 65.7 |
| SFFS | 50.0 | | 46.4 | | 93.0 | | 11 | F15 | | 63.2 |
| NCA | 50.0 | | 58.6 | | 87.5 | | 19 | F20 | | 65.4 |
| MRMR | 45.8 | | 37.0 | | 87.1 | | 6 | F20 | | 56.6 |
| **Thr = 0.80** |  | |  | |  | |  |  | |  |
| - | 45.0 | | 52.2 | | 82.4 | | 32 | - | | 61.7 |
| SFS | 50.0 | | 55.6 | | 83.6 | | 5 | F15 | | 62.9 |
| SBS | 50.0 | | 62.1 | | 80.6 | | 19 | F25 | | 64.6 |
| SFFS | 50.0 | | 55.3 | | 86.4 | | 13 | F15 | | 63.7 |
| NCA | 50.0 | | 62.5 | | 80.0 | | 14 | F15 | | 64.5 |
| MRMR* | 50.0 | | 67.9 | | 91.6 | | 12 | F1 | | 66.5 |
| **Thr = 0.85** |  | |  | |  | |  |  | |  |
| - | 38.2 | | 33.3 | | 82.7 | | 43 | - | | 51.4 |
| SFS* | 50.0 | | 57.1 | | 85.7 | | 6 | F20 | | 64.3 |
| SBS | 35.4 | | 29.6 | | 80.0 | | 42 | F30 | | 48.4 |
| SFFS | 50.0 | | 37.9 | | 83.6 | | 23 | F15 | | 57.2 |
| NCA | 45.8 | | 37.0 | | 87.1 | | 30 | F25 | | 56.6 |
| MRMR | 50.0 | | 7.8 | | 87.1 | | 18 | F1 | | 61.8 |
| Feat. = feature; Thr = correlation threshold; Bal_acc = balanced accuracy; HL_acc = hypopharynx and larynx accuracy; OC_acc = oral cavity accuracy; #Feat = number of features used and correspondent frequency threshold. Mean is the average of Bal_acc, HL_acc and OC_acc; * = feature set that obtained the best mean compared to the other feature sets from the same correlation threshold. | | | | | | | | | | |

**Combination of P2-S1 and P2-S2: radiomic model**

**Table S8:** Results obtained by combining P2-S1 and P2-S2 using radiomic features. The first two columns identifies the correlation threshold and feature selection method that were chosen in each step to be included in the model development.

| **Thr & feat. selection** | | **Accuracy** | | | |
| --- | --- | --- | --- | --- | --- |
| **P2-S1** | **P2-S2** | **HL (%)** | **OC (%)** | **Oph (%)** | **NPh (%)** |
| 0.75 - | 0.75 - | 59.3 | 92.7 | 83.3 | 87.1 |
|  | 0.80 MRMR | 61.5 | 96.4 |  |  |
|  | 0.85 SFS | 46.2 | 92.7 |  |  |
| 0.80 SBS | 0.75 - | 58.6 | 77.8 | 85.8 | 89.8 |
|  | 0.80 MRMR | 69.0 | 83.8 |  |  |
|  | 0.85 SFS | 55.2 | 87.5 |  |  |
|  | 0.75 - | 59.3 | 84.9 | 89.2 | 94.0 |
| 0.85 NCA | 0.80 MRMR | 66.7 | 79.1 | 89.0 |  |
|  | 0.85 SFS | 51.9 | 82.6 | 89.2 |  |
| Thr & feat. selection = correlation threshold and feature selection method; HL = hypopharynx and larynx; OC = oral cavity; OPh = oropharynx; NPh = nasopharynx. | | | | | |
|  | | | | | |

**P2: Clinical model performance**

**Table S9:** Results obtained from P2 using clinical features and training Linear SVM.

| **Clinical model** | **Bal_acc (%)** | **Accuracies** | | | |
| --- | --- | --- | --- | --- | --- |
|  |  | **OPh (%)** | **NPh (%)** | **HL (%)** | **OC (%)** |
| P2-S1 | 74.7 | 62.7 | 61.0 | - | - |
| P2-S2 | 33.4 | - | - | 77.2 | 44.2 |
| Bal_acc = balanced accuracy; HL = hypopharynx and larynx; OC = oral cavity; OPh = oropharynx; NPh = nasopharynx. | | | | | |

**P2: Radiomic-clinical model performance**

**Table S10**: Results obtained from P2-S1 using radiomic and clinical features and training Linear SVM.

| **Feat. Selection** | **Bal_acc(%)** | | **OPh_acc (%)** | | **NPh_acc(%)** | | **#Feat** | | | **Mean** |
| --- | --- | --- | --- | --- | --- | --- | --- | --- | --- | --- |
| **Thr = 0.75** |  |  | |  | |  | | |  |  |
| - | 84.8 | | 70.4 | | 84.3 | | 27 | - | | 79.8 |
| SFS | 85.3 | | 67.1 | | 84.5 | | 9 | F20 | | 79.0 |
| SBS | 84.7 | | 70.2 | | 82.4 | | 18 | F25 | | 79.1 |
| SFFS | 82.7 | | 62.7 | | 82.1 | | 14 | F15 | | 75.9 |
| NCA* | 86.3 | | 69.6 | | 84.5 | | 16 | F25 | | 80.1 |
| MRMR | 82.7 | | 65.4 | | 79.3 | | 18 | F1 | | 75.8 |
| **Thr = 0.80** |  | |  | |  | |  |  | |  |
| - | 86.4 | | 71.7 | | 88.1 | | 35 | - | | 82.1 |
| SFS | 87.1 | | 71.3 | | 89.1 | | 33 | F1 | | 82.5 |
| SBS* | 87.1 | | 72.5 | | 89.1 | | 30 | F20 | | 82.9 |
| SFFS | 85.1 | | 66.5 | | 86.4 | | 16 | F15 | | 79.3 |
| NCA | 88.2 | | 69.8 | | 90.2 | | 8 | F25 | | 82.7 |
| MRMR | 62.2 | | 65.0 | | 64.2 | | 12 | F1 | | 71.6 |
| **Thr = 0.85** |  | |  | |  | |  |  | |  |
| - | 88.1 | | 70.4 | | 92.9 | | 46 | - | | 83.8 |
| SFS | 88.2 | | 71.3 | | 93.1 | | 43 | F1 | | 84.2 |
| SBS* | 88.8 | | 71.5 | | 95.2 | | 33 | F25 | | 85.2 |
| SFFS | 89.3 | | 70.4 | | 93.3 | | 26 | F15 | | 84.4 |
| NCA | 89.1 | | 69.2 | | 95.2 | | 12 | F25 | | 84.5 |
| MRMR | 80.1 | | 66.3 | | 74.8 | | 5 | F20 | | 73.7 |
| Feat. = feature; Thr = correlation threshold; Bal_acc = balanced accuracy; OPh_acc = oropharyx accuracy; NPh_acc = nasopharynx accuracy; #Feat = number of features used and correspondent frequency threshold. Mean is the average of Bal_acc, OPh_acc and NPh_acc; * = feature set that obtained the best mean compared to the other feature sets from the same correlation threshold. | | | | | | | | | | |

**Table S11**: Results obtained from P2-S2 using radiomic and clinical features and training Linear SVM.

| **Feat. Selection** | **Bal_acc(%)** | | **HL_acc (%)** | | **OC_acc(%)** | | **#Feat** | | | **Mean** |
| --- | --- | --- | --- | --- | --- | --- | --- | --- | --- | --- |
| **Thr = 0.75** |  |  | |  | |  | | |  |  |
| - | 40.0 | | 73.0 | | 69.0 | | 27 | - | | 60.7 |
| SFS | 40.6 | | 67.3 | | 73.6 | | 9 | F20 | | 60.5 |
| SBS* | 40.3 | | 78.0 | | 75.6 | | 18 | F25 | | 64.6 |
| SFFS | 41.2 | | 78.2 | | 72.1 | | 14 | F15 | | 63.8 |
| NCA | 39.1 | | 69.3 | | 69.5 | | 16 | F25 | | 59.3 |
| MRMR | 40.3 | | 1.4 | | 71.4 | | 18 | F1 | | 61.1 |
| **Thr = 0.80** |  | |  | |  | |  |  | |  |
| - | 39.8 | | 71.4 | | 70.1 | | 35 | - | | 60.4 |
| SFS | 41.2 | | 70.4 | | 66.8 | | 33 | F1 | | 59.5 |
| SBS | 40.0 | | 71.0 | | 65.0 | | 30 | F20 | | 58.7 |
| SFFS | 42.7 | | 70.3 | | 73.1 | | 16 | F15 | | 62.0 |
| NCA | 35.0 | | 57.4 | | 62.4 | | 8 | F25 | | 51.6 |
| MRMR* | 46.3 | | 75.5 | | 75.6 | | 12 | F1 | | 65.8 |
| **Thr = 0.85** |  | |  | |  | |  |  | |  |
| - | 42.3 | | 65.3 | | 66.2 | | 46 | - | | 57.9 |
| SFS | 38.8 | | 63.0 | | 65.0 | | 43 | F1 | | 55.6 |
| SBS* | 43.7 | | 66.7 | | 80.0 | | 33 | F25 | | 59.0 |
| SFFS | 41.2 | | 69.0 | | 83.6 | | 26 | F15 | | 57.9 |
| NCA | 41.7 | | 73.6 | | 87.1 | | 12 | F25 | | 56.9 |
| MRMR | 35.4 | | 53.3 | | 87.7 | | 5 | F20 | | 52.7 |
| Feat. = feature; Thr = correlation threshold; Bal_acc = balanced accuracy; HL_acc = hypopharynx and larynx accuracy; OC_acc = oral cavity accuracy; #Feat = number of features used and correspondent frequency threshold. Mean is the average of Bal_acc, OPh_acc and NPh_acc; * = feature set that obtained the best mean compared to the other feature sets from the same correlation threshold. | | | | | | | | | | |

**Combination of P2-S1 and P2-S2: radiomic-clinical model**

**Table S12:** Results obtained by combining P2-S1 and P2-S2 using radiomic and clinical features. The first two columns identify the correlation threshold and feature selection method that were chosen in each step to be included in the model development.

| **Thr & feat. selection** | | **Accuracy** | | | |
| --- | --- | --- | --- | --- | --- |
| **P2-S1** | **P2-S2** | **HL (%)** | **OC (%)** | **OPh (%)** | **NPh (%)** |
| 0.75 NCA | 0.75 SBS | 77.2 | 75.6 | 69.4 | 84.5 |
|  | 0.80 MRMR | 75.2 | 75.6 |  |  |
|  | 0.85 SBS | 67.3 | 67.0 |  |  |
| 0.80 SBS | 0.75 SBS | 79.0 | 75.6 | 72.5 | 88.3 |
|  | 0.80 MRMR | 75.0 | 75.6 |  |  |
|  | 0.85 SBS | 67.0 | 67.0 |  |  |
|  | 0.75 SBS | 78.8 | 75.4 | 71.5 | 95.2 |
| 0.85 SBS | 0.80 MRMR | 75.8 | 75.4 |  |  |
|  | 0.85 SBS | 67.7 | 66.7 |  |  |
| Bal_acc = balanced accuracy; HL = hypopharynx and larynx; OC = oral cavity; OPh = oropharynx; NPh = nasopharynx. | | | | | |
|  | | | | | |

**Comparing the obtained results from P2**

**Table S14**: Comparison of the results obtained from P2 by applying radiomic features, clinical features and radiomic with clinical features. The accuracies computed in this table are the average of the accuracies presented in Table S8 and Table S12, i.e., for each class the average is computed regardless of the correlation threshold. The results obtained by applying only clinical features are the same ones presented in Table S9 without computing the average.

|  | **Average accuracy** | | | | |
| --- | --- | --- | --- | --- | --- |
| **Model** | **HL (%)** | **OC (%)** | **OPh (%)** | | **NPh (%)** |
| Radiomic | 58.6 | 86.2 | 87.2 | 90.4 | |
| Clinical | 62.7 | 61.0 | 77.2 | 44.2 | |
| Radiomic-clinical | 73.6 | 72.7 | 71.1 | 89.3 | |
| Bal_acc = balanced accuracy; HL = hypopharynx and larynx; OC = oral cavity; OPh = oropharynx; NPh = nasopharynx. | | | | | |

**SVM model parameters for the best model in P2-S1 0.80 SBS and in P2-S2 0.75 SBS**

**Table S13**: Parameters and the correspondent values used to train the SVM models for the combination of 0.80 SBS in P2-S1 and 0.75 SBS in P2-S2.

| **Parameter** | **P2-S1 and P2-S2** |
| --- | --- |
| Kernel Function | linear |
| Kernel Scale | auto |
| Box Constrain | 1 |
| Standardize | true |
| Polynomial Order | [] |
